# Pancreatic cancer tumor organoids exhibit subtype-specific differences in metabolic profiles

**DOI:** 10.1186/s40170-024-00357-z

**Published:** 2024-10-03

**Authors:** Hassan A. Ali, Joanna M. Karasinska, James T. Topham, Danisha Johal, Steve Kalloger, Andrew Metcalfe, Cassia S. Warren, Anthony Miyagi, Lan V. Tao, Maya Kevorkova, Shawn C. Chafe, Paul C. McDonald, Shoukat Dedhar, Seth J. Parker, Daniel J. Renouf, David F. Schaeffer

**Affiliations:** 1 Pancreas Centre BC, Vancouver, BC Canada; 2 Department of Biochemistry and Molecular Biology, University of British Columbia, Vancouver, BC Canada; 3 Department of Integrative Oncology, BC Cancer Research Institute, Vancouver, BC Canada; 4 BC Children’s Hospital Research Institute, Vancouver, BC Canada; 5 Division of Medical Oncology, BC Cancer, Vancouver, BC Canada; 6 Department of Medicine, University of British Columbia, Vancouver, BC Canada; 7 Division of Anatomic Pathology, Vancouver General Hospital, Vancouver, BC Canada; 8 Department of Pathology and Laboratory Medicine, University of British Columbia, Vancouver, BC Canada

**Keywords:** PDAC, Organoids, PDAC tumor subtype, Glycolysis, OXPHOS, MPC1, Metabolic profiling

## Abstract

**Background:**

Pancreatic ductal adenocarcinoma (PDAC) is a highly aggressive disease characterized by complex metabolic rewiring that enables growth in changing nutrient availability and oxygen conditions. Transcriptome-based prognostic PDAC tumor subtypes, known as ‘basal-like’ and ‘classical’ subtypes are associated with differences in metabolic gene expression including genes involved in glycolysis. Tumor subtype-specific metabolism phenotypes may provide new targets for treatment development in PDAC, but their functional relevance has not been fully elucidated. We aimed to investigate differences in metabolic profiles and transcriptomes in tumor models derived from patients with basal-like and classical tumors.

**Methods:**

Patient-derived organoids (PDOs) were established from tumor biopsies collected from patients with metastatic PDAC, including three PDOs from basal-like and five PDOs from classical tumors. Metabolic analyses included assessment of differences in metabolic activity using Seahorse Glycolysis and Mito Stress tests and ^13^C-glucose metabolites tracing analysis. In order to investigate the influence of mitochondrial pyruvate transport on metabolic differences, PDOs were treated with the mitochondrial pyruvate carrier 1 (MPC1) inhibitor UK-5099. Prognostic relevance of MPC1 was determined using a tumor tissue microarray (TMA) in resectable, and proteomics profiling in metastatic PDAC datasets. Whole genome and transcriptome sequencing, differential gene expression and gene set enrichment analyses were performed in PDOs.

**Results:**

Metastatic PDAC PDOs showed subtype-specific differences in glycolysis and oxidative phosphorylation (OXPHOS). Basal-like tumor-derived PDOs had a lower baseline extracellular acidification rate, but higher glycolytic reserves and oxygen consumption rate (OCR) than classical tumor-derived PDOs. OCR difference was eliminated following treatment with UK-5099. In the ^13^C-glucose metabolites tracing experiment, a basal-like tumor PDO showed lower fractions of some M + 2 metabolites but higher sensitivity to UK-5099 mediated reduction in M + 2 metabolites than a classical tumor PDO. Protein level analyses revealed lower MPC1 protein levels in basal-like PDAC cases and association of low MPC1 levels with clinicopathologic parameters of tumor aggressiveness in PDAC. PDO differential gene expression analyses identified additional subtype-specific cellular pathways and potential disease outcome biomarkers.

**Conclusions:**

Our findings point to distinct metabolic profiles in PDAC subtypes with basal-like tumor PDOs showing higher OXPHOS and sensitivity to MPC1 inhibition. Subtypes-specific metabolic vulnerabilities may be exploited for selective therapeutic targeting.

**Supplementary Information:**

The online version contains supplementary material available at 10.1186/s40170-024-00357-z.

## Background

Pancreatic ductal adenocarcinoma (PDAC) is currently the third leading cause of cancer-related deaths and has a five-year survival rate of 13% [1]. PDAC is characterized by the presence of oncogenic *KRAS* mutations in about 90% of tumors, in addition to high prevalence of mutations or copy losses in tumor suppressor genes *TP53*,* CDKN2A*, and *SMAD4* [2]. These and other oncogenic events induce metabolic dysregulation in the tumor microenvironment contributing to the aggressiveness and chemoresistance of PDAC [3, 4]. Glycolysis is one of the most studied processes associated with metabolic reprogramming in cancer cells and involves energy production through the cytosolic degradation of glucose in a series of enzymatic steps ending in the production of pyruvate [5]. In addition to glycolysis helping cells survive hypoxic conditions, cancer cells maintain high glycolysis rates even in the presence of oxygen, which offers a growth advantage to invasive cells [6, 7]. PDAC cells can survive and proliferate under oxygen-deprived conditions by activating hypoxia-induced factors and maintaining high rates of glycolysis and oxidative mitochondrial metabolism, the latter enabled by utilizing glutamine as an alternative precursor for mitochondrial aspartate production [8, 9]. Shifting from glucose to glutamine and fatty acid oxidation also allows proliferating cells to preserve mitochondrial ATP-linked respiration under conditions of reduced mitochondrial pyruvate uptake, such as that caused by inhibition of mitochondrial pyruvate complex (MPC) [10]. MPC is formed by hetero-oligomers of subunits mitochondrial pyruvate carrier 1 and 2 (MPC1 and MPC2) and mediates pyruvate transport into the mitochondria [10]. A decrease in MPC activity, driven by reduced expression of *MPC1*, promotes glycolysis and tumor progression in several cancer types [11, 12].

PDAC tumors can be stratified into molecular subtypes based on distinct genomic and transcriptomic features, which have been shown to associate with prognosis [10, 13–16] and, for a small subset of tumors, confer sensitivity to targeted therapies [17–19]. PDAC transcriptome subtypes broadly fall into the dichotomous classification of basal-like (or squamous) and classical tumors, with basal-like (squamous) tumors being associated with worse prognosis [20] and, genomic profiles including enrichment for chromatin modifier gene mutations, complete loss of *CDKN2A* and mutant *KRAS* imbalance [14, 21]. We previously demonstrated that tumor expression of genes involved in glycolysis and cholesterol synthesis pathways correlated with ‘basal-like’ and ‘classical’ gene expression, respectively [22]. Tumors with a ‘glycolytic’ gene profile had the worst prognosis and exhibited lower *MPC1* expression [4], suggesting that specific tumor metabolic pathways may contribute to prognostic differences between PDAC subtypes.

Here, we further investigated the metabolic mechanisms underlying subtype functional heterogeneity and analyzed glycolytic and respiratory activity in patient-derived organoid (PDO) models derived from basal-like and classical metastatic PDAC tumors. We observed subtype-specific differences in glucose utilization between PDOs, including in response to MPC1 inhibition, highlighting the role of reprogrammed glucose metabolism as a factor contributing to PDAC tumoral heterogeneity and differences in subtype aggressiveness.

## Methods

### Organoid culture media

Advanced DMEM/F-12 (Thermo Fisher Scientific, 12634010) supplemented with 1X GlutaMAX (Thermo Fisher Scientific, 35050061), 10mM HEPES (Thermo Fisher Scientific, 15630080), 100𝜇g/mL primocin (Invivogen, ant-pm-1), 1X B-27 supplement (Thermo Fisher Scientific, 17504044), 1.25mM N-Acetyl-L-cysteine (Sigma-Aldrich, A9165), 10nM Gastrin-I (Sigma-Aldrich, G9020), 100ng/mL Recombinant Human FGF-10 (Peprotech, 100 − 26), 0.5𝜇M A 83 − 01 (Tocris, 2939), 10𝜇M Y-27632 (Tocris, 1254), 10𝜇M Nicotinamide (Sigma-Aldrich, N0636) and Recombinant Human EGF (Peprotech, AF-100-15) was mixed 1:1 with Wnt-3a/R-Spondin1/Noggin conditioned medium (ATCC, 3276) to create complete growth medium (CGM). Advanced DMEM/F-12 was supplemented with 5 mg/mL Collagenase II (Thermo Fisher Scientific, 12604013) to create digestion medium. Seahorse XF DMEM (Agilent, 103575-100) was supplemented with 1X GlutaMax to create Glycolysis Stress Test assay medium. Seahorse XF DMEM was supplemented with 10mM Seahorse XF glucose solution (Agilent, 103577-100), 1X GlutaMax and 1mM Seahorse XF 100mM pyruvate solution (Agilent, 103578-100) to create Mito Stress Test assay medium. SILAC Advanced DMEM/F-12 Flex Media (Thermo Fisher Scientific, A2494301) was supplemented with 91.25𝜇g/mL L-Lysine (Sigma-Aldrich, L5501), 147.5𝜇g/mL L-Arginine (Sigma-Aldrich, A5006) and 3.15 mg/mL [U]-^13^C -D-Glucose (Sigma-Aldrich, 389374) to create SILAC assay media.

### Establishment and culture of patient derived organoids

Organoids were established and grown using a previously described protocol by the Tuveson group [23]. PDAC tumor tissue from metastatic lesions from the liver were obtained as fine needle biopsy cores. Necrotic regions and blood vessels were excised, tissues were then minced and incubated in digestion media at 37^o^C and 5% CO_2_ for 12–16 h. Dissociated cells were seeded into growth factor reduced Matrigel (Corning, 354230) domes and cultured in CGM at 37^o^C and 5% CO_2_. CGM was replaced every 4 days and organoids were passaged upon reaching 80% dome confluency. To passage, CGM was replaced with 1X TrypLE Express (Thermo Fisher Scientific, 12604013), domes were mechanically fragmented, and wells were incubated at 37^o^C and 5% CO_2_ for 30 min. Dissociated cells were seeded into Matrigel domes and cultured in CGM at 37^o^C and 5% CO_2_.

### Whole genome and transcriptome sequencing

PanGen (NCT02869802) tumor tissue and organoid samples were subjected to whole genome and transcriptomic sequencing as previously described [22]. Briefly, fresh tumor biopsies and matched normal (blood) were sequenced at a target depth of 80X and 40X, respectively. Libraries had reads trimmed to 75 base pairs (bp) and were aligned (hg19; GRCh37-lite) using BWA-mem v0.7.6a [24] with default parameters and duplicate reads were marked using sambamba v0.5.5 [25] with default parameters. RNA sequencing had a target depth of 200 million reads and reads were trimmed to 75 bp and aligned (GRCh37-lite) using STAR v2.7.3 [26] with parameters: -chimSegmentMin 20 -outSAMmultNmax 1 -outSAMstrandField intronMotif -outFilterIntronMotifs RemoveNoncanonical; and duplicate read were marked using PicardTools v2.17.3.

Raw reads counts were assigned to Ensembl 75 genes using Subread v1.4.6 [27], normalized for library depth and gene size (RPKM) and log10-transformed. All sequencing was performed at the Canada’s Michael Smith Sciences Centre at BC Cancer.

### Mass spectrometry based proteomics

MS-based proteomics sequencing of tumor biopsy samples from PanGen patients diagnosed with metastatic PDAC (*n* = 45) was performed using the SP3-CTP pipeline as previously described [28]. MPC1 and MPC2 protein data were extracted from the dataset.

### Subtype classification

PDAC patient tumor samples were classified into Moffit PurIST subtypes as outlined by previous studies [22, 28]. Briefly, PurIST subtyping was based on expression of 8 gene pairs to estimate basal-like probability. PurIST scores (probability of basal-like) assigned to each sample were used to assign a basal-like (score > 0.5) or classical (score < 0.5) subtype. PDOs were assigned subtypes of the biopsies from which they were derived. For NanoString platform-based PurIST subtyping, available formalin-fixed, paraffin embedded whole tumor tissue sections matching the tissue microarray (TMA) cases were processed and RNA was sequenced as described previously [29].

### Seahorse extracellular acidification rate (ECAR) and oxygen consumption rate (OCR) analysis

ECAR and OCR were measured using a Seahorse XFe96 analyzer (Agilent). XFe96 microplate wells were coated with 1:10 Matrigel diluted in CGM and microplates were incubated at room temperature for 1.5 h. Cells were seeded at 10,000 cells/well and grown at 37^o^C 5% CO_2_ for 96 h, followed by incubation with DMSO (Sigma-Aldrich, D2650) or 5𝜇M UK-5099 (Sigma-Aldrich, 5048170001) for 48 h. Wells were washed and media was replaced with glycolysis stress assay medium or mito stress assay medium. The microplate was then incubated at 37^o^C and atmospheric CO_2_ for one hour during which well confluency was measured using an Incucyte analyzer (Sartorius). Glycolysis rates were measured in response to sequential injections of glucose (10mM), oligomycin (5𝜇M) and 2-DG (25mM) (Agilent, 103020-100) and respiratory rates were measured in response to sequential injections of oligomycin (5𝜇M), FCCP (2𝜇M) and Rotenone/Antimycin-A (0.5𝜇M) (Agilent, 103015-100). All measurements were normalized to well confluency and all data were processed with a modified version of Seahorse Explorer (SHORE) [30].

### TMA analysis

A TMA containing 0.6 mm tissue cores from 252 resected PDAC tumors with non-missing clinico-pathologic and outcome data was utilized for this study. MPC1 immunohistochemistry was performed on the Ventana Discovery Ultra research staining system (Ventana Medical Systems). In brief, 4 micron tissue sections underwent 64 min of CC1 (Cell Conditioning 1, Ventana Medical Systems) before MPC1 antibody (Sigma-Aldrich, HPA045119) was applied for 2 h with no heat at 1:100 titration. The HQ-HRP detection system (Ventana Medical Systems Inc) was used to visualize the protein. Centralized immunohistochemistry was conducted using the Ventana XT platform (Ventana Medical Systems) according to the standard procedures. An H-score was calculated as the product of percent of epithelial cells staining positive and a subjective assessment of staining intensity utilizing a three tier ordinal scale. Resultant H-Scores ranged from 0 to 300. The core from each duplicate set with the highest H-Score was considered the score for each case. Disease specific survival time dependent recursive partitioning was used to derive a cut-point for high and low expressers of MPC-1 respectively.

### Stable-isotope tracing

PDOs were seeded at 100,000 cells per well in CGM at 37^o^C and 5% CO_2_ for 96 h. Media was then replaced with SILAC assay media (with ^13^C -D-Glucose) containing DMSO or 5𝜇M UK-5099 in triplicates for 48 h. The supernatant for each well was transferred to eppindorf tubes and flash frozen in liquid nitrogen. To obtain cell pellet, Matrigel domes were resuspended in 1000𝜇L 1X ice-cold PBS (Thermo Fisher Scientific, 10010023) and transferred to 15mL falcon tubes. Tubes were centrifuged at 500 RCF and 4^o^C for 5 min and the supernatant was discarded. To remove any residual Matrigel, 500𝜇L TryPLE solution was added and tubes were incubated in a 37^o^C water bath for 15 min. The resulting cell pellet was flash frozen in liquid nitrogen until metabolite extraction.

For metabolite extraction, buffer containing 500𝜇L methanol (-20^o^C) (VWR, BDH20864.400), 200𝜇L HPLC grade water (4^o^C) (Sigma-Aldrich, 270733), and 1ng/𝜇L norvaline (Alfa Aesar, B23444) was added to tubes with PDO pellets. Components were transferred to screw top vials containing 500𝜇L chloroform (Omnisolv, CX1054-1) and 0.5 mm silica disruption beads (RPI) and homogenized via BeadBlaster 24 bead-mill homogenizer (Benchmark Scientific) at 4^o^C for 3 cycles of high energy impact at 6 m/s. Entire tube contents were transferred to a new Eppendorf tube where aqueous and inorganic layers were separated by cold centrifugation for 15 min. 450𝜇L of the aqueous layer containing polar metabolites was transferred to a new tube and evaporated using a SpeedVac (Thermo Scientific) prior to derivatization and GC-MS analysis. For media extraction, 5𝜇L of conditioned media was extracted in 250uL of 80% methanol solution containing 1ng/𝜇L norvaline and dried using a SpeedVac. Derivatization of polar metabolites was conducted using MOX-tBDMCS derivatization as previously described [31]. Derivatized samples were analyzed by GC-MS using a DB-35 column interfaced with an Agilent 8890 Gas Chromatograph (GC) coupled to Agilent 5977B Series MSD mass spectrometer (MS).

### Differential expression, enrichment and correlation analysis

A differential gene expression analysis was performed between PDOs from basal-like (*n* = 3) and classical (*n* = 5) biopsies using PDO transcriptomic data. Raw mRNA counts of protein-coding genes (*n* = 18,287) were used as input for DEA by DESeq2 [32] with default parameters and the design formula: ~ purist_call. The reference group was set to classical. Downstream gene set enrichment analysis was performed using fgsea [33]. Unfiltered ranked Log2 Fold Change values from the DEA were used as input along with gene sets with sizes between 20 and 200 genes (*n* = 23,104) from the molecular signatures data base (MsigDB [34], downloaded June 2022). P values for both DEA and GSEA were subjected to Benjamini-Hochberg multiple test correction. Genes from the MsigDB dataset WP_AEROBIC_GLYCOLYSIS were tested for correlation (spearman) between biopsies and matched PDOs.

### Statistical analysis

A Student’s t-test was performed for comparisons between two groups. Wilcoxon mean rank-sum tests were used for two-group comparisons of continuous variables. Spearman correlations were used to test correlation between two continuous variables. Categorical comparisons with two-levels were computed using Fisher’s exact test, categorical comparisons with greater than two levels were quantified with the likelihood-ratio X^2^ test. All p values were subjected to Benjamini-Hochberg multiple test correction when applicable and a threshold adjusted p value of 0.05 was used for statistical significance. Survival analysis were performed using the Kaplan-Meier method with differences calculated using the Log-Rank statistic. Seahorse experiments were analyzed using Prism (v9.0.0, GraphPad). All transcriptomic, genomic and metabolomic analysis were performed using R v4.2.2. Results are expressed as mean ± SEM unless otherwise indicated.

## Results

### PDOs from basal-like and classical tumors show different glycolytic and oxidative phosphorylation profiles

**Fig. 1:**
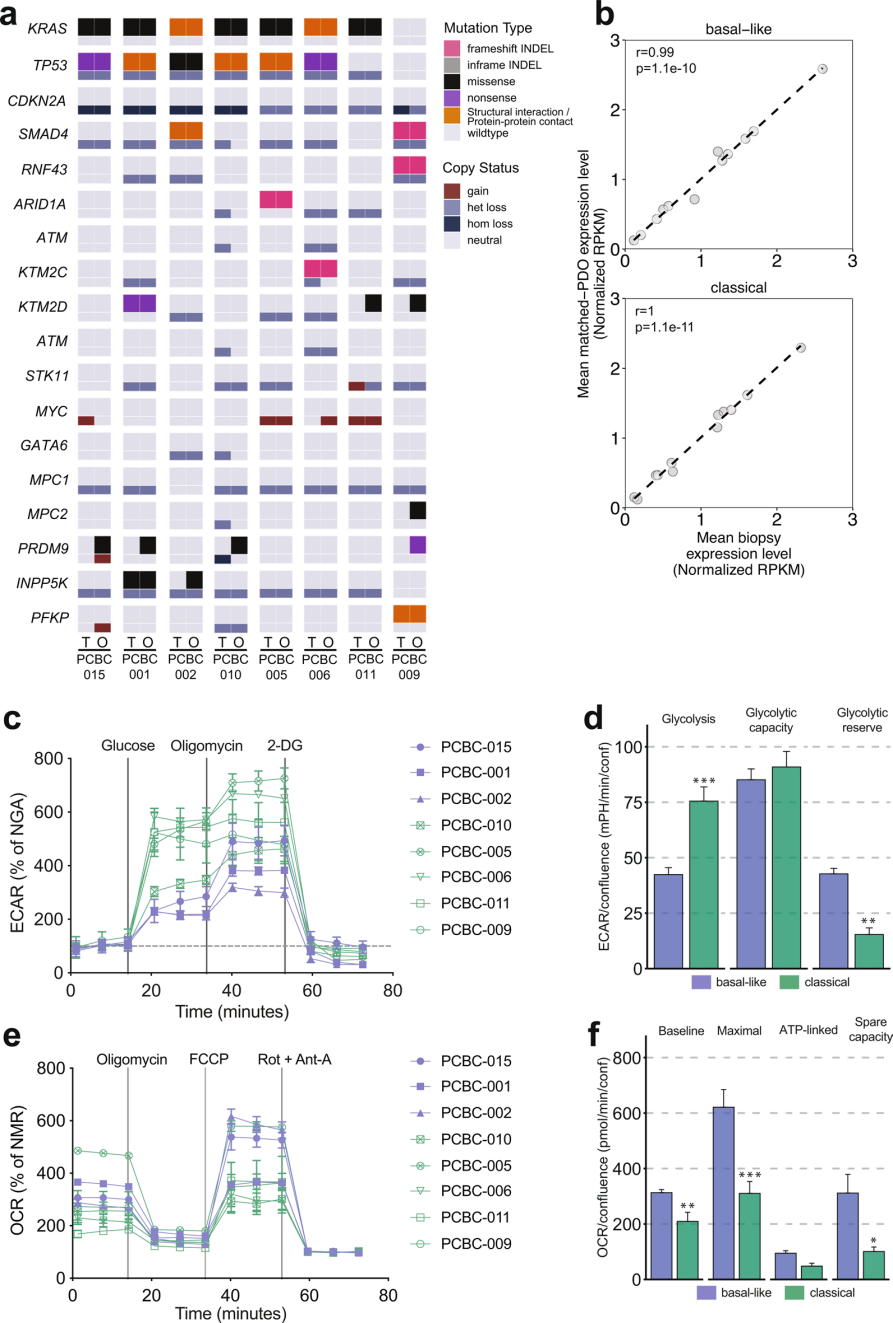
PDOs capture genomic heterogeneity of patient tumors and display subtype specific alterations in metabolism. (**a**) Oncoprint comparing somatic mutations (SNV/indel) and copy number variation (CNV) events in frequently mutated and metabolic genes between patient tumors (T) and matched PDOs (O). (**b**) Scatter plot depicting correlation in expression of glycolytic genes between patient tumors and derived PDOs with basal-like or classical subtypes (spearman correlation; Benjamini–Hochberg multiple test correction). (**c**) Extracellular acidification rate (ECAR) curves of PDOs after injections of glucose, oligomycin and 2-DG during a Glycolysis Stress Test. There are multiple phases recorded, baseline glycolysis refers to ECAR after glucose and before oligomycin injection; maximum glycolysis refers to ECAR after oligomycin injection and before 2-DG injection; and non-glycolytic acidification (NGA) refers to ECAR before injection of glucose or after injection of 2-DG. (**d**) Bar plots of grouped comparison between PDOs from basal-like and classical tumors showing differences in glycolysis measurements. (**e**) Oxygen consumption rate (OCR) curves of PDOs after injections of oligomycin, FCCP and Rotenone + Antimycin-A during a Mito Stress Test. Multiple assay phases were recorded, baseline respiration refers to OCR prior to oligomycin injection; maximum respiration refers to OCR after FCCP injection and before Rotenone + Antimycin-A injection; ATP-linked respiration is the difference between basal respiration and OCR after oligomycin injection and before FCCP injection; and non-mitochondrial respiration (NMR) refers to OCR after injection of Rotenone + Antimycin-A. (**f**) Bar plots of grouped comparison between PDOs from basal-like and classical tumors showing differences in OXPHOS measurements. *p *<* 0.05, **p *<* 0.01, ***p *<* 0.001 (Students t-test)

We previously reported a correlation between expression of basal-like PDAC subtype genes and genes involved in glycolysis [35], suggesting that basal-like tumors may have a higher capacity for increasing glucose utilization to meet energy demands. We used PDO models derived from genomically annotated metastatic PDAC liver biopsies (*n* = 8, demographic information in Supplementary Table 1) in order to investigate differences in tumor metabolic activity between basal-like and classical subtypes at the cellular level. Genomic analysis showed that PDOs largely recapitulated the oncogenic driver gene mutations of their origin biopsies, but demonstrated some differences in alterations in less frequently mutated genes such as *PRDM9* (Fig. 1a), a finding that is in line with other studies [36, 37]. An earlier study by our group reported the prognostic stratification of PDAC tumors based on genes involved in glycolysis [22]. Transcriptome analysis demonstrated that glycolytic gene expression was highly correlated between PDOs and their matched tumor biopsies in both basal-like (*r* = 0.99, *p* = 1.1e-10) and classical (*r* = 1, *p* = 1.1e-11) tumor subgroups (Fig. 1b and Supplementary Fig. S1), suggesting that PDOs are relevant in vitro models to facilitate glycolytic analysis in PDAC. We measured rates of glycolysis and oxidative phosphorylation (OXPHOS) in PDOs utilizing Seahorse XFe96 glycolytic and mitochondrial Stress Tests. Extracellular acidification rate (ECAR) and oxygen consumption rate (OCR) were used to assess glycolysis and OXPHOS activity, respectively. ECAR measurements following glucose administration in Seahorse Glycolytic Stress Tests (Fig. 1c) showed that in comparison to classical tumor PDOs, basal-like tumor PDOs had lower baseline glycolysis (*p* < 0.0001) rates but higher glycolytic reserves (*p* < 0.0001), measured as the difference between glycolytic capacity and baseline glycolysis (Fig. 1d).This suggests that classical PDOs operate near maximum glycolytic potential, whereas basal-like PDOs may be able to upregulate glycolysis in response to mitochondrial respiratory stress. Analysis of OXPHOS rates measured in the Seahorse Mito Stress Test (Fig. 1e) showed higher baseline (*p* = 0.008), maximal (*p* = 0.0006) and spare capacity rates (*p* = 0.01) in PDOs from basal-like tumors (Fig. 1f). The OCR: ECAR ratios were higher in basal-like PDOs (Supplementary Fig. S2). Overall, these findings suggest that in addition to higher glycolytic reserves, basal-like tumor PDOs maintain higher levels of ATP generation from OXPHOS, which could be one of the mechanisms that enable basal-like tumors to adapt to metabolic changes in the tumor microenvironment.

### MPC1 inhibition by UK-5099 shifts metabolic profile of PDAC PDOs

**Fig. 2:**
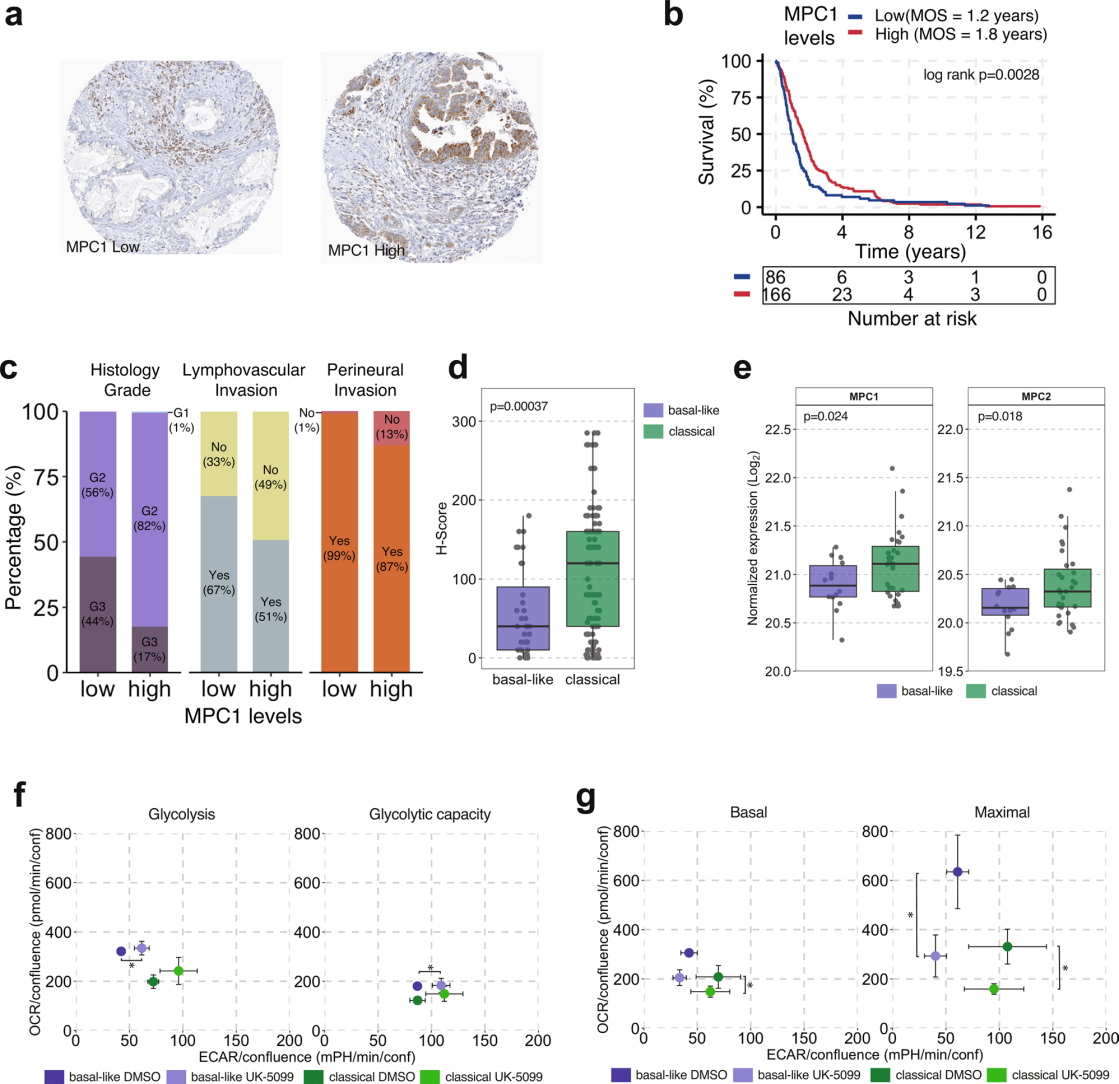
Reduced MPC1 levels are associated with disease aggressiveness and shifts in metabolic activity in PDOs. (**a**) Immunohistochemistry images showing low (left) and high (right) MPC1 protein levels in PDAC TMA (**b**) Kaplan-Meier survival analysis of patient tumors with high and low MPC1 protein levels. (**c**) Bar plots illustrating the association of MPC1 levels with histological grade (*p* < 0.0001), lymphovascular invasion (*p* = 0.01) and perineural invasion (*p* = 0.0009). (**d**) Box plots illustrating H-scores between basal-like and classical subtypes in TMA of resected PDAC tumors. (**e**) Box plots showing expression of MPC1 (left) and MPC2 (right) protein in biopsies of metastatic PDAC patient tumors (*n* = 45). (**f**) Scatter plots illustrating OCR and ECAR changes in basal-like and classical PDOs treated with a MPC1 inhibitor (UK-5099) following glucose injection (left) and oligomycin injection (right) during a Glycolysis Stress Test. Basal-like PDOs had increased baseline glycolysis levels upon inhibition of MPC1. (**g**) Scatter plots illustrating OCR and ECAR changes in basal-like and classical PDOs treated with a MPC1 inhibitor (UK-5099) during basal respiration (left) and post FCCP injection (right) during a Mito Stress Test. Basal-like PDOs had increased OCR during basal respiration whereas both basal-like and classical PDOs had increased OCR upon inhibition of MPC1. All ECAR and OCR data points are represented as mean ± SEM. *p *<* 0.05, **p *<* 0.01, ***p *<* 0.001

The mitochondrial pyruvate carrier MPC is a heterodimer of MPC1 and MPC2, and mediates the transport of pyruvate into the mitochondria where it is used as a substrate in the tricarboxylic acid (TCA) cycle and OXPHOS [35]. Reduction in MPC1 levels and activity contribute to cancer progression by promoting epithelial to mesenchymal transition (EMT) [38] or increasing resistance to chemotherapy [39], and lower *MPC1* expression is associated with the PDAC glycolytic subtype, a marker of poor prognosis [22]. To explore the association with MPC1 protein levels and tumor aggressiveness parameters, we interrogated a clinically annotated tumor tissue microarray comprising the tumor epithelium component of 252 resected PDAC cases collected by the Gastrointestinal Biobank at the Vancouver General Hospital, using a MPC1 antibody (Fig. 2a). An immunohistochemistry H-score of 55 was determined to stratify the patient cohort into two groups designated as low MPC1 (H-score < 55, *n* = 86) and high MPC1 (H-score ≥ 55, *n* = 166), with a difference in median disease-specific survival of 6.8 months (*p* = 0.007, Fig. 2b). In addition to shorter survival, low MPC1 levels were associated with higher frequency of grade 3 tumors (44% vs. 17% in high MPC1 cases, *p* < 0.0001, Fig. 2c), lymphovascular invasion (67% vs. 51%, *p* = 0.01) and perineural invasion (99% vs. 87%, *p* = 0.0009). Nanostring-based PurIST subtyping data were available for 163 TMA cases. Basal-like tumors had lower MPC1 H-scores than classical tumors (*p* = 0.00037, Fig. 2d). We further validated this finding in the PanGen dataset of metastatic PDAC cases. MS-based proteomic profiling of tumor tissue samples (*n* = 45) demonstrated lower levels of both MPC subunits MPC1 (*p* = 0.024) and MPC2 (*p* = 0.018) in basal-like tumors (Fig. 2e). These findings from two orthogonal cohorts provide further validation for the prognostic value of MPC1 levels, and suggest that a decrease in MPC levels contributes to tumor aggressiveness in PDAC.

To investigate whether MPC1 inhibition increases glycolysis or reduces OXPHOS in tumor cells, we measured ECAR and OCR in PDOs treated with the MPC1 small molecule inhibitor UK-5099. No significant changes were observed in cell growth in response to UK-5099 (Supplementary Fig. S3). In the Glycolytic Stress Test, MPC1 inhibition led to a small increase in ECAR in PDOs from basal-like tumors (p *=* 0.03, Fig. 2f), and a trend towards an increase in PDOs from classical tumors (p *=* 0.08). In the Mito Stress Test, UK-5099 treatment reduced OCR in classical PDOs during basal respiration (p *=* 0.04*)*, and in both basal-like and classical PDOs in the maximum respiration step (p *=* 0.02 and *p* = 0.03, respectively, Fig. 2g). Hence, inhibition of MPC1 resulted in a small increase in glycolytic activity in PDOs from both tumor subtypes and attenuated OCR in basal-like PDOs to levels similar to classical PDOs under non-treated conditions. These data suggest that higher OXPHOS in basal-like tumors may be facilitated by mitochondrial pyruvate uptake via MPC1 despite lower MPC1 protein levels.

Distinct MPC configurations were shown to have higher pyruvate transport rates under respiratory conditions in yeast [40], suggesting that high pyruvate transport activity may be maintained by specific MPC oligomer composition and nutrient conditions, even in the presence of lower MPC1 levels.

### Inhibition of mitochondrial pyruvate transport impacts generation of 13C-glucose-derived metabolites in PDAC PDOs

**Fig. 3:**
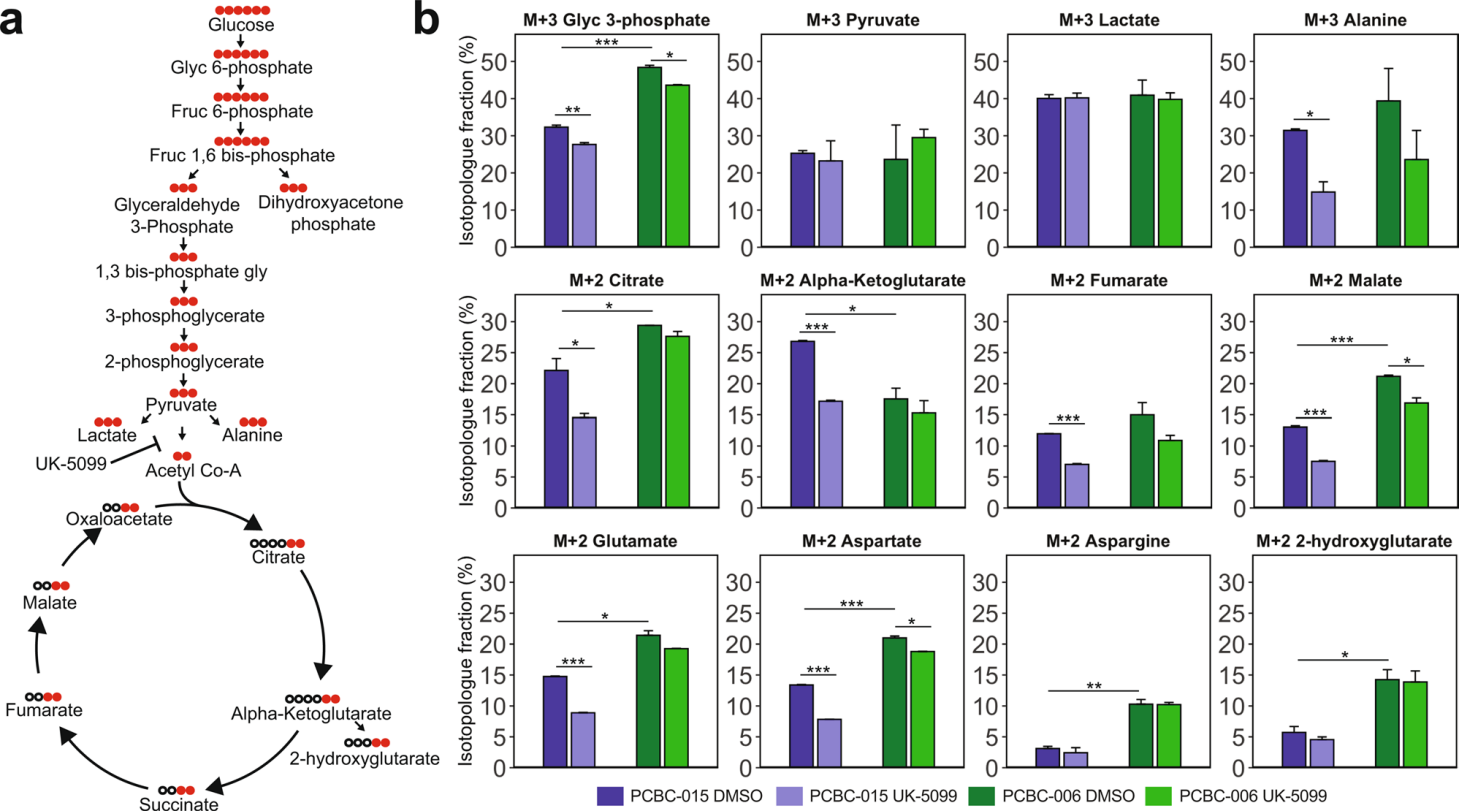
UK-5099 leads to a subtype-specific decrease in ^13^C labelling of glycolysis and TCA cycle metabolites. (**a**) Illustration of metabolites generated during glycolysis and TCA cycle with carbon backbone lengths. Black dots represent ^12^C and red dots ^13^C. (**b**) Bar plots showing fractions of labelled (M + 2/M + 3) carbon pool for glycolysis and TCA cycle metabolites along with select amino acids; following DMSO and UK-5099 (5𝜇M) treatment in PCBC-015 (basal-like) and PCBC-006 (classical). *p < 0.05, **p < 0.01, ***p < 0.001 (students t-test)

Lower ECAR and higher OCR baseline values in PDOs from basal-like tumors suggest that these PDOs may utilize a higher fraction of glycolysis-derived pyruvate to feed the mitochondrial TCA cycle. To investigate the differences in glucose utilization for glycolytic and TCA cycle metabolites, we selected one classical (PCBC-006) and one basal-like (PCBC-015) PDO model for a ^13^C-glucose tracing study (Fig. 3a). Analysis of glycolytic intermediates in cell pellets showed higher M + 3 fraction of labelled Glyc-3-phosphate (Glyc3P) in PCBC-006 (Fig. 3b, *p* < 0.0001), and a small reduction in labelled Glyc3P in response to UK-5099 in both PCBC-006 (p *=* 0.01) and PCBC-015 (*p* = 0.003). No differences were observed in the labelled M + 3 pyruvate or lactate fractions between the PDOs or in response to MPC1 inhibition in the cell pellets. In the supernatant, PCBC-006 had a higher fraction of M + 3 labelled pyruvate (*p* < 0.001) and lactate (*p* < 0.001) than PCBC-0015 (Supplementary Fig. S4), consistent with the observed higher baseline glycolysis in classical PDOs; following UK-5099, labelled fractions of pyruvate and lactate were increased in both PDOs. UK-5099 reduced M + 3 labelled alanine fraction in PCBC-015 (p *<* 0.05), which may be due either to the diversion of labelled glycolytic intermediates towards pyruvate or lactate, or differences in glucose utilization for the generation of mitochondrial alanine between PDOs.

In the M + 2 fractional pool, PCBC-015 had a lower fraction of labelled TCA metabolites including citrate (*p* = 0.02) and malate (*p* < 0.001) but a higher fraction of M + 2 alpha-ketoglutarate (αKG, *p* = 0.03) than PCBC-006. These differences may be due to lower rates of conversion of αKG to glutamate or 2-hydroxyglutarate as reflected in their lower M + 2 fractions in PCBC-015, or higher conversion rates of citrate into lipid precursors or M + 2 malate in PCBC-006. PCBC-006 had higher fractions of M + 2 aspartate (p *<* 0.001) and M + 2 asparagine (*p* < 0.01), suggesting higher rates of conversion of glucose-derived oxaloacetate to aspartate in the classical tumor PDO. Upon treatment with UK-5099, the basal-like tumor derived PCBC-015 showed reduced M + 3 fraction of Glyc3P (p *<* 0.01), alanine (*p* < 0.05), the TCA metabolites M + 2 citrate (*p* < 0.05), αKG (*p* < 0.001), fumarate (*p* < 0.001) and malate (*p* < 0.001), as well as M + 2 glutamate and aspartate. Small reductions in M + 3 Glyc3P, M + 2 malate and aspartate were observed in PCBC-006 following UK-5099 treatment. The higher sensitivity to UK-5099 observed in the basal-like tumor derived PCBC-015 suggests that this PDO may have greater reliance on pyruvate from glycolysis to feed the TCA cycle and is more susceptible to MPC1 inhibition.

### Differential gene expression analysis of PDOs identifies cellular pathway alterations and potential PDAC biomarkers

**Fig. 4:**
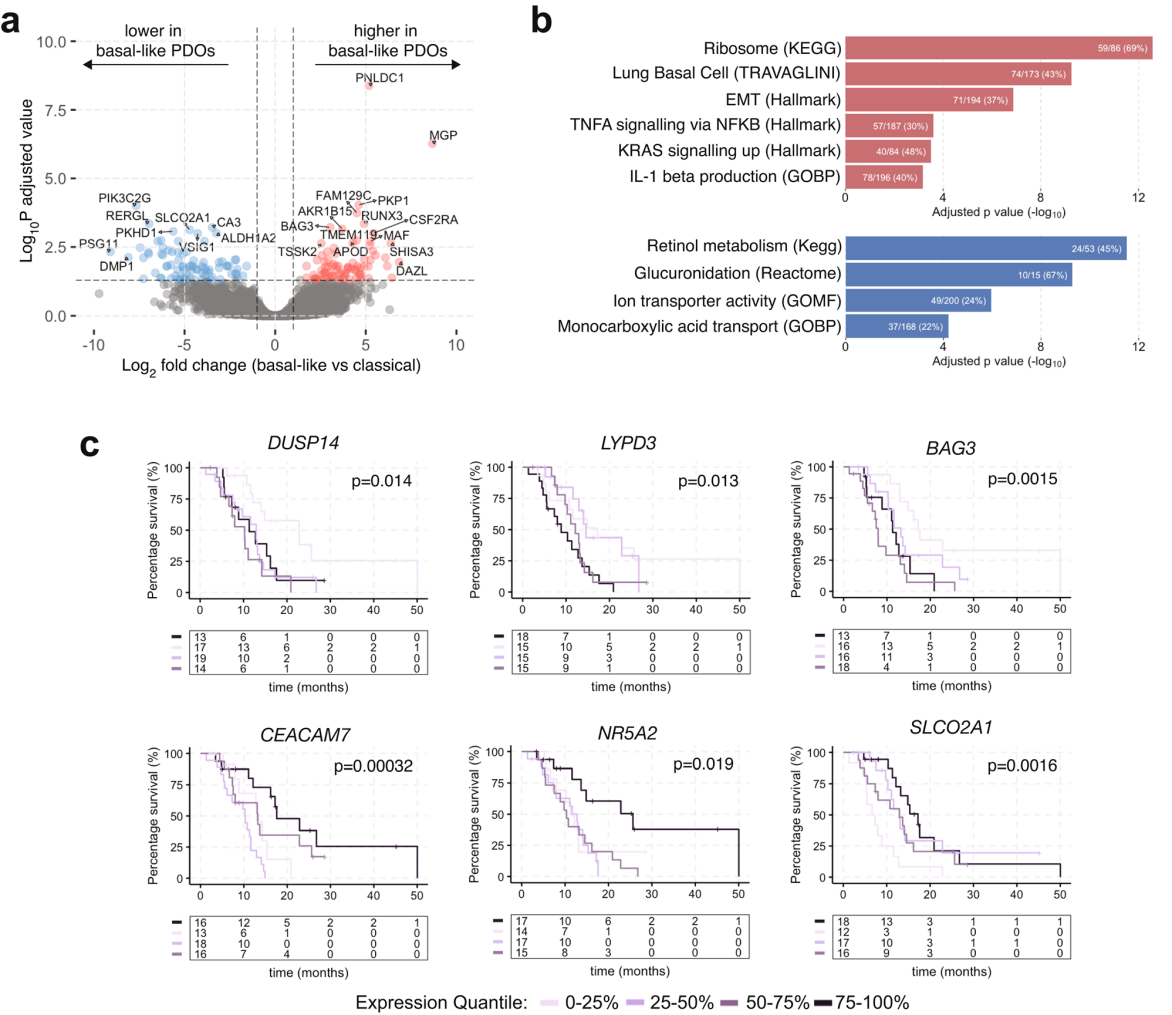
Differential gene expression analysis between PDOs from basal-like and classical tumors. (**a**) Volcano plot of differentially expressed genes between PDOs from basal-like and classical tumors. Expression in basal-like PDOs is shown in reference to classical PDOs. (**b**) Bar plots showing gene set enrichment analysis performed on up (top; red) and down (bottom; blue) regulated genes in basal-like PDOs compared to classical PDOs. Genes enriched in basal-like PDOs are belong to pathways that associate with aggressive tumors (**c**) Kaplan-Meier survival curves depicting association of median overall survival with gene expression quantiles in a cohort of PDAC metastatic biopsies (PanGen, *n* = 69)

The majority of PDAC transcriptome subtype classifications have been derived from bulk tumors comprising tumor epithelium and other tumor microenvironment compartments [20]. To assess for any potential tumor cell intrinsic genes and related pathways that may elucidate distinct molecular or functional differences between basal-like and classical tumors, or to identify novel prognostic biomarkers, we performed a differential gene expression analysis (DEA) between PDOs derived from basal-like versus classical patient tumors. The DEA identified 78 and 80 genes with higher and lower expression in basal-like tumors, respectively (*p* < 0.05, absolute log2fold change (L2FC) > 2, Fig. 4a and Supplementary Table 2). None of the 50 top genes stratifying the Moffitt et al. [15] basal-like and classical subtypes reached significant differences in the DEA following multiple comparison test correction. This may be due to the small sample size, as a few Moffitt genes showed differences in expression in individual comparisons (data not shown). Furthermore, the local tumor microenvironment influences the PDAC cell state and subtype [41], and the absence of the physiological tumor microenvironment may contribute to transcriptomic changes in cell culture. Gene set enrichment analysis (GSEA), using 23,104 pathways, showed that genes upregulated in PDOs from basal-like tumors were enriched for gene sets involved in RNA translation, epithelial-to-mesenchymal transition, KRAS and cytokine signalling (Fig. 4b and Supplementary Table 3). Genes downregulated in PDOs from basal-like tumors were enriched for gene sets involved in monocarboxylic and bile acid transport, ion channel transport, retinol metabolism and glucuronidation.

Among upregulated genes in PDOs derived from basal-like tumors were genes that have been associated with tumor progression or cell survival including *BAG3* [42] (log-rank *p* = 0.0015), *DUSP14* [43] (log-rank *p* = 0.014), and *LYPD3* [44] (log-rank *p* = 0.013). Higher expression of these genes was associated with shorter survival in patients with metastatic PDAC (Fig. 4c). DEA genes that were down-regulated in basal-like PDOs with prognostic impact in the PanGen patient cohort included *SLCO2A1*, *CEACAM7* and *NR5A2*. *SLCO2A1* encodes a bidirectional prostaglandin transporter PGT and its low expression is associated with worse prognosis in gastric and breast cancers [45, 46]. The expression of *CEACAM7* encoding a cell adhesion protein is downregulated in colorectal carcinoma [47]. *NR5A2*, also known as *LRH1* is a transcriptional factor involved in regulation of cell metabolism, acinar cell differentiation and pancreas inflammation [48, 49]. Overall, the differential gene expression and pathway analysis, and prognostic impact of individual genes highlights their potential utility as biomarkers or therapeutic targets in PDAC.

## Discussion

Genome and transcriptome based subtyping of PDAC has identified prognostic and treatment sensitivity-predictive molecular profiles, but the clinical actionability of PDAC molecular subtypes remains limited. Only about 10% of patients with PDAC have tumors that harbor mutations in *BRCA1/2* or *PALB2*, and *NRG1* gene fusions, and may benefit from targeted therapy with cisplatin, olaparib or tyrosine kinase inhibitors, respectively [17, 19, 50]. Transcriptome focused basal-like (squamous) and classical subtyping is a commonly used stratification approach due to its prognostic impact [14, 20] but currently there is still limited knowledge of distinct cellular mechanisms underlying subtype differences. Gene expression analysis and metabolite profiling suggest that basal-like and classical tumors may utilize different metabolic programs [22, 51–53]. Here, we report that organoids derived from basal-like tumors have higher rates of mitochondrial respiration than classical tumor-derived organoids, which may be dependent on glycolysis-derived pyruvate.

In addition to high rates of aerobic glycolysis observed in cancer cells, where lactate is generated from glucose even in the presence of oxygen, cancer cells also maintain high levels of OXPHOS, which enhances their migratory and metastatic potential [54–56]. Ablation of mutant *KRAS* in a PDAC mouse model led to tumor regression followed by relapse facilitated by a fraction of surviving tumor cells with higher dependency on OXPHOS [57]. Higher glucose oxidation in tumors has been reported in patients with lung adenocarcinoma [58]. We observed higher oxygen consumption rates in PDOs from basal-like tumors, suggesting that these organoids may represent tumor cells with a more aggressive phenotype than the classical tumor PDOs. Despite having lower baseline glycolysis rates, basal-like PDOs showed the ability to increase their glycolytic rate following inhibition of ATP synthase activity with oligomycin, suggesting that they can shift towards higher glycolysis when needed. This points to heterogeneity in metabolic plasticity in PDAC subtypes where basal-like tumors may be more adaptable to changes in the tumor metabolic conditions and oxygen supply. Hypoxia is often associated with PDAC, and basal-like tumors have been reported to have higher hypoxia and glycolytic gene expression scores than classical tumors [22, 59]. Future experiments using both normal and hypoxic cell culture conditions, and oxidative fuel restriction conditions, should further elucidate the metabolic heterogeneity of PDOs.

MPC-mediated mitochondrial pyruvate uptake is thought to dampen the effects of glycolysis by re-directing pyruvate to the mitochondrial TCA cycle, and away from its conversion to lactate [12]. *MPC1* is downregulated in several cancers and in the glycolytic PDAC subtype associated with poor prognosis [12, 22]. Using two independent cohorts of PDAC (resectable and metastatic), we validated the association of low MPC1 and clinicopathologic parameters of aggressive PDAC, including lower MPC protein subunit expression in basal-like tumors, further supporting the notion that MPC1 may inhibit tumor metastatic potential. Interestingly, the MPC1 inhibitor UK-5099 had a small effect on ECAR and no effect on the fraction of ^13^C-glucose-derived lactate in PDOs from either basal-like or classical tumors, suggesting a lack of major impact of UK-5099 on glycolysis. However, UK-5099 caused a marked decrease in maximal OCR with a more pronounced effect in the basal-like tumor PDOs, and reduced ^13^C-glucose-derived fraction of TCA cycle intermediates in a basal-like PDO. This indicates that glucose oxidation is driving OXPHOS in basal-like PDAC and could be a potential target for treatment. Metformin, an inhibitor of OXPHOS, failed to establish efficacy in a phase 3 randomized trial in hormone receptor positive breast cancer patients. However, metformin was associated with longer survival in patients with *ERBB2* amplified tumors [60], and *ERBB2* amplification is associated with altered mitochondrial activity [61]. Phenformin, an analogue of metformin, increased gemcitabine sensitivity in PDAC cells with high OXPHOS [62], suggesting that metformin may benefit a subset of PDAC patients with high OXPHOS tumors. In addition to pyruvate, glucose-derived alanine can enter the mitochondria via a yet unidentified transporter, where it is converted to pyruvate [63]. Lower response to MPC1 inhibition in the Seahorse and ^13^C-glucose tracing experiments suggests that classical tumor PDOs may use an alternative transport pathway for TCA cycle intermediates. Overall our findings imply that MPC may regulate at least some glycolytic and OXPHOS activity in PDAC. However other nutrients including lipids and amino acids may also impact the metabolic differences between PDAC subtypes [64, 65]. Comprehensive knowledge of metabolic gene and activity profiles in basal-like and classical tumors may enable the development of subtype-targeted metabolism-based therapeutics.

In addition to targeting distinct metabolic pathways in PDAC subtypes, the development of new treatments will benefit from the knowledge of oncogenic, cell signalling and other pathways that differentiate between the subtypes. PDO transcriptome analysis can help identify tumor cell-intrinsic genes and pathways enriched in specific subtypes. We leveraged PDO transcriptomic data from eight PDOs to identify additional potential gene sets and biomarkers associated with basal-like and classical gene programs. Several of the differentially expressed genes in PDOs were prognostic in a patient cohort, highlighting their potential significance in disease progression.

In conclusion, we provide evidence for distinct metabolic alterations in PDAC organoids derived from basal-like and classical patient tumors, with basal-like tumor cells exhibiting a metabolic profile that may facilitate aggressiveness. The limitation of our study is the small sample size which impacts the power of statistical analysis and does not fully represent the diversity of patient population. Future collaborative efforts aimed at generating sharable cross-centre PDO databanks capturing the patient population diversity, PDAC stages and subtypes would be beneficial to continuous progress in pancreatic cancer research. Our data are compatible with the notion that targeting subtype-specific metabolic vulnerabilities maybe a promising approach in the treatment of PDAC.

## Electronic supplementary material

Below is the link to the electronic supplementary material.

Supplementary Material 1

Supplementary Material 2

Supplementary Material 3

## Data Availability

No datasets were generated or analysed during the current study.

## Abbreviations

bp: Base pair
DEA: Differential expression analysis
ECAR: Extracellular acidification rate
EMT: Epithelial to mesenchymal transition
GSEA: Gene set enrichment analysis
MPC: Mitochondrial pyruvate complex
MPC1: Mitochondrial pyruvate carrier 1
MPC2: Mitochondrial pyruvate carrier 2
OCR: Oxygen consumption rate
OXPHOS: Oxidative phosphorylation
PDAC: Pancreatic ductal adenocarcinoma
PDO: Patient derived organoid
TCA: Tricarboxylic acid cycle
