# Supplementary material for: Pancreatic cancer tumor organoids exhibit subtype-specific differences in metabolic profiles
Source: Cancer Metab. 2024 Oct 3;12:28. doi: 10.1186/s40170-024-00357-z (PMC11448267; doi:10.1186/s40170-024-00357-z)
Supplement: Supplementary file 1 — Supplementary Material 1 [file 40170_2024_357_MOESM1_ESM.docx]

**Supplementary Figures and Table**

**
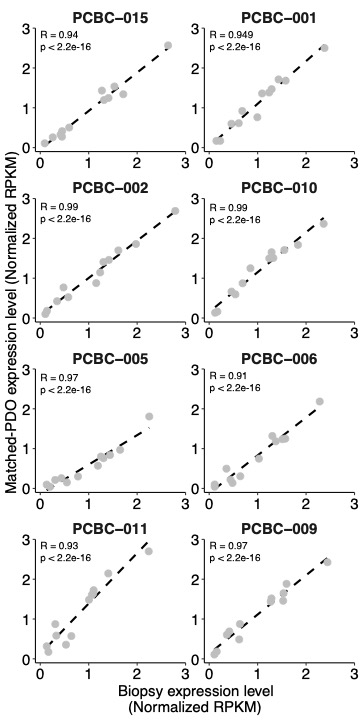
**

**Supplementary Figure S1: Expression of glycolytic genes is correlated between biopsies and matched PDOs.** Scatter plot depicting expression of glycolytic genes between patient tumours and matched PDOs.


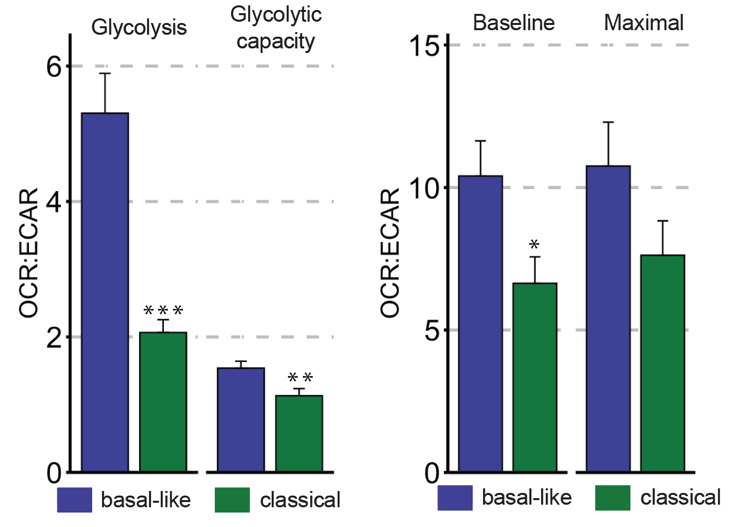


**Supplementary Figure S2: Basal-like PDOs showed increased OCR:ECAR during basal respiration.** Bar graphs showing OCR:ECAR during baseline and maximal respiration phases of the mito stress test in basal-like and classical PDOs. **p<* 0.05


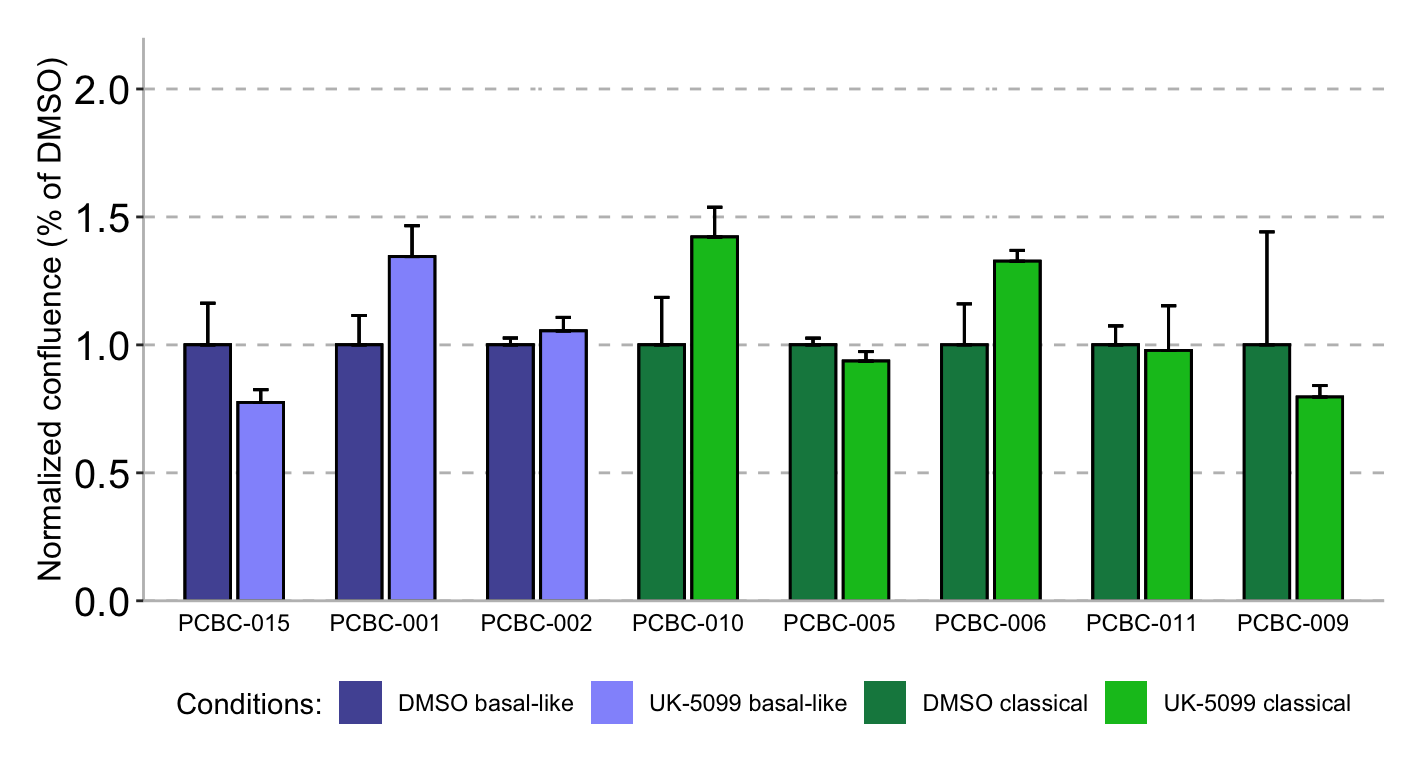


**Supplementary Figure S3: Treatment with UK-5099 does not impact growth in PDOs.** Bar graphs of confluence taken prior to seahorse assay illustrating no significant changes in confluence between DMSO and UK-5099 in all PDOs.

**Supplementary Figure S4: Classical PDOs had increased ^13^C labelling in M+3 Pyruvate and M+3 Lactate than basal-like PDOs in the supernatant.** Bar graphs showing fractions of M+3 carbon pool for pyruvate and lactate in the supernatant following DMSO and UK-5099 (5𝜇M) treatment in PCBC-015 (basal-like) and PCBC-006 (classical). ****p<* 0.001


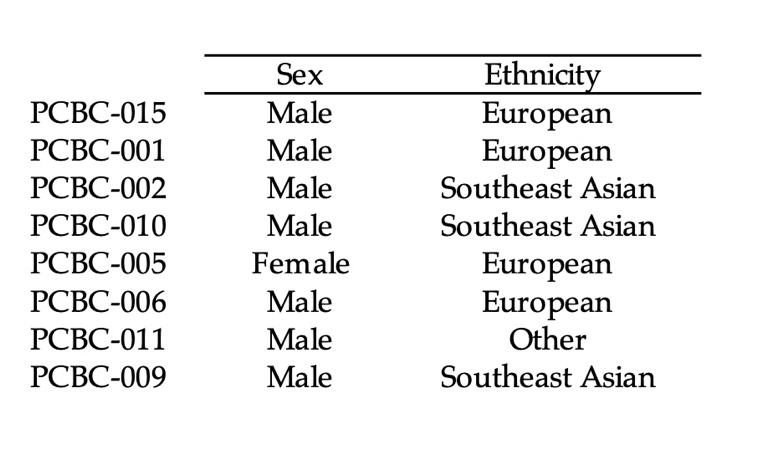


**Supplementary Table 1: Sex and Ethnicity distribution of PDO cohort**
